# B7-H3 at the crossroads between tumor plasticity and colorectal cancer progression: a potential target for therapeutic intervention

**DOI:** 10.1007/s10555-023-10137-8

**Published:** 2023-09-28

**Authors:** Elizabeth Varghese, Samson Mathews Samuel, Aranka Brockmueller, Mehdi Shakibaei, Peter Kubatka, Dietrich Büsselberg

**Affiliations:** 1 Department of Physiology and Biophysics, Weill Cornell Medicine-Qatar, Education City, Qatar Foundation, P.O. Box 24144, Doha, Qatar; 2 Chair of Vegetative Anatomy, Institute of Anatomy, Faculty of Medicine, Ludwig-Maximilians-University Munich, Pettenkoferstr. 11, 80336 Munich, Germany; 3 Department of Histology and Embryology, Jessenius Faculty of Medicine, Comenius University in Bratislava, Mala Hora 4, 036 01 Martin, Slovakia

**Keywords:** Colorectal cancer, B7-H3, Immunomodulation, Tumor plasticity, immune checkpoint, Drug resistance

## Abstract

B7-H3 (B7 homology 3 protein) is an important transmembrane immunoregulatory protein expressed in immune cells, antigen-presenting cells, and tumor cells. Studies reveal a multifaceted role of B7-H3 in tumor progression by modulating various cancer hallmarks involving angiogenesis, immune evasion, and tumor microenvironment, and it is also a promising candidate for cancer immunotherapy. In colorectal cancer (CRC), B7-H3 has been associated with various aspects of disease progression, such as evasion of tumor immune surveillance, tumor-node metastasis, and poor prognosis. Strategies to block or interfere with B7-H3 in its immunological and non-immunological functions are under investigation. In this study, we explore the role of B7-H3 in tumor plasticity, emphasizing tumor glucose metabolism, angiogenesis, epithelial-mesenchymal transition, cancer stem cells, apoptosis, and changing immune signatures in the tumor immune landscape. We discuss how B7-H3-induced tumor plasticity contributes to immune evasion, metastasis, and therapy resistance. Furthermore, we delve into the most recent advancements in targeting B7-H3-based tumor immunotherapy as a potential approach to CRC treatment.

## Introduction

Colorectal cancer (CRC) is the third most common type of cancer globally, and it is estimated that there were more than 1.9 million new cases and 930,000 deaths worldwide in 2020 [1, 2]. By 2040, the incidence of CRC is predicted to increase by 63% and the number of deaths by 73.4% [1]. CRC-related deaths are common in more advanced age groups between 50 and 74 years. The occurrence of CRC has been linked to lifestyle factors such as low consumption of fruits and vegetables and high consumption of meat, and other modifiable risk factors include obesity, lack of exercise, high alcohol intake, and frequent consumption of processed food. Hence, CRC is largely preventable through lifestyle modifications, spreading awareness, and early screening programs. Depending on the stage of the disease, clinical management of CRC includes surgery, chemotherapy, and radiation. However, these treatments reach their limits in the advanced stages of CRC, where metastases often occur in the liver and peritoneum [3].

To address the recurrence, metastasis, and treatment failure in CRC, it is essential to understand, discuss, and shed light on the significance of tumor heterogeneity in connection with tumorigenesis, tumor progression, and immune evasion. Tumor heterogeneity refers to spatial and temporal differences in the composition and function of cells that have evolved during tumorigenesis. This can be due to multiple factors, and one among them is the cumulative effect of random mutations contributing to genetic heterogeneity as explained by the models of “clonal evolution,” “stem cell,” and “big bang” model [4]. Beyond the genetic factors, metabolic heterogeneity within the tumor is related to cancer cells having different preferences and dependencies for their energy requirements. Depending upon the oxygen gradient and proximity to vascular tissues, some cancer cells depend on glycolysis.

In contrast, other cancer cells preferably depend on oxidative phosphorylation (OXPHOS) or glutamine metabolism to meet their energy needs for cell survival and proliferation [5]. This metabolic plasticity within the tumor niche is an energy-efficient mechanism for cancer cell survival and proliferation. It shapes the immune landscape of tumors by making cancer cells less vulnerable to the innate immune system (immune evasion). Recent advances in research on the B7 family of proteins have identified their multifaceted role in tumor plasticity through their engagement in reprogramming cellular metabolism, reshaping the immune landscape, and enriching stemness and senescence, thereby contributing to relapse and therapy resistance. In this review, we focus on the transmembrane protein B7 homology 3 protein (B7-H3) or Cluster of Differentiation 276 (CD276) that belongs to the B7 family of immune checkpoint proteins. This protein is present in all active cells involved in the tumor microenvironment (TME) and we discuss how it contributes to CRC progression at the cellular level and its various clinical implications.

## B7-H3: an overview

B7-H3 is a member of the B7 family of immune checkpoint proteins encoded by the gene CD276 gene. The B7 family consists of seven structurally related proteins [B7.1 (CD80), B7.2 (CD86), inducible costimulatory ligand (ICOS-L), programmed death-1 ligand (PD-L1), programmed death-2 ligand (PD-L2), B7-H3, and B7-H4] that binds to the receptors belonging to CD28 family of proteins present on the lymphocytes (T- and B-lymphocytes) and other non-lymphoid cells [6]. Furthermore, new proteins belonging to the B7 family were identified, bringing the total number of members in B7 family to eleven [7]. B7-H3 is expressed in several tissues, such as the uterus, liver, heart, prostate, and colon, and low levels in the brain, kidney, lung, and skeletal muscle. They are broadly expressed at the RNA level, but their protein expression is relatively low in normal tissues, possibly due to a post-transcriptional mechanism [8]. Several reports have highlighted that the overexpression of B7-H3 in various cancers, including colorectal, cervical, and prostate, could contribute to metastasis, therapy resistance, poor prognosis, and reduced overall survival in various cancers [9–11].

**Fig. 1:**
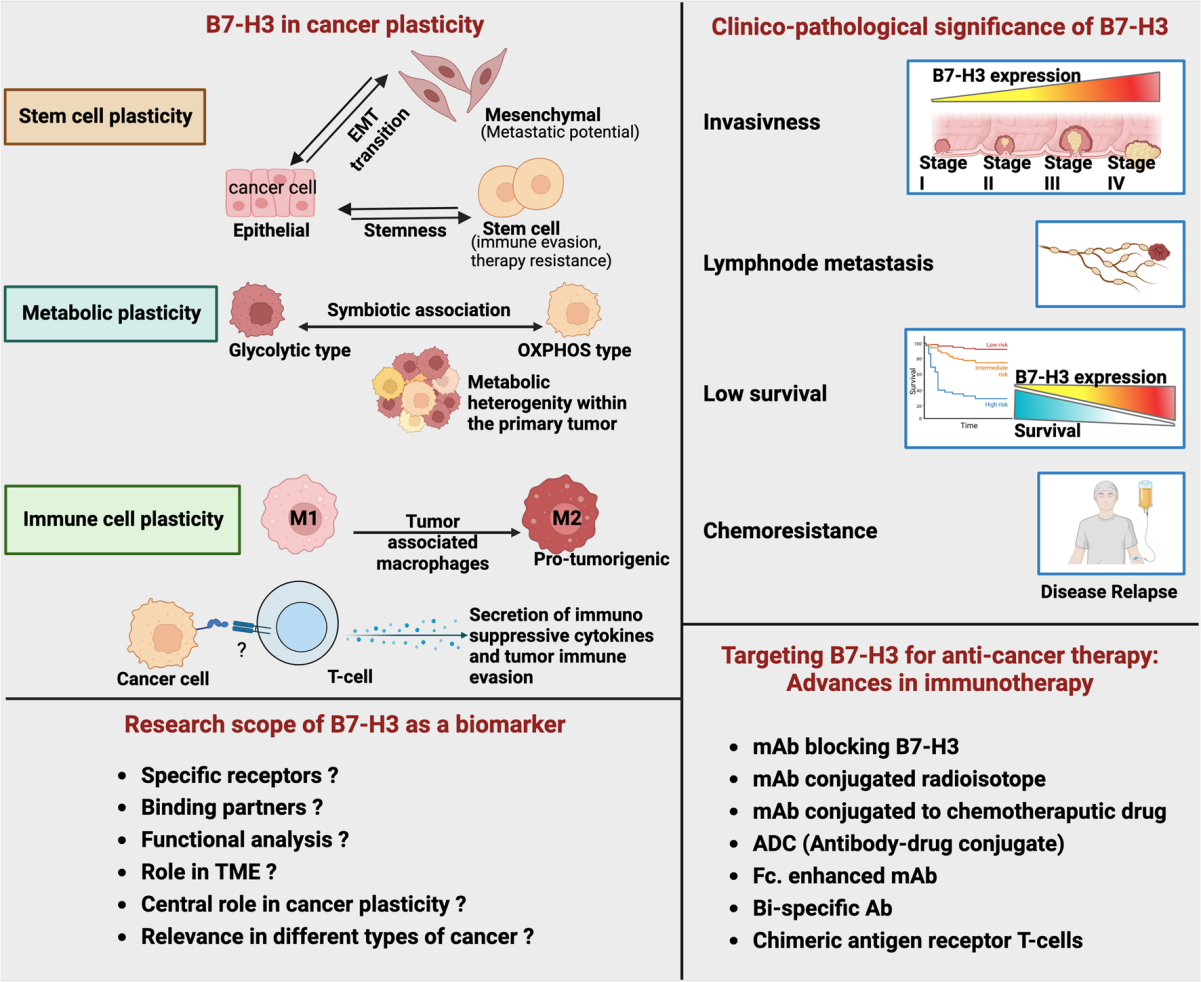
Overview of B7-H3. B7 homolog 3 protein (B7-H3), or (CD276), an immune checkpoint protein, is overexpressed in tumors and are barely expressed in normal tissue. It can induce pro-oncogenic traits. Cancer characteristics such as adhesion, invasion, migration, and metastasis are affected by B7-H3 expression. Apart from this, it contributes to immune evasion. Upregulated B7-H3 expression is reported in clinical samples and is associated with metastasis, therapy resistance and low survival. B7-H3 is a potential target for anticancer therapy as it can modulate different cancer hallmarks and can regulate innate and adaptive immunity as well. Immune based therapies are the latest trend in cancer treatment. Multiple modalities targeting B7-H3 is underway and outcomes from the clinical trials are giving promising results. Nevertheless, unresolved questions open opportunities for further exploring this unique immune checkpoint protein. Created with BioRender.com. mAb, monoclonal antibody; Ab, antibody; TME, tumor microenvironment

Tumor plasticity refers to cancer cell’s ability to switch between different physiological states, creating a potential to proliferate, disseminate, and evade immune recognition (Fig. 1). This feature aids the cancer cell to thrive and resist chemotherapy and contributes to relapse. Understanding the underlying mechanism of tumor cell plasticity will enable to design more efficient treatment methods and, ultimately, patient survival. More research in this field has identified a firm link connecting B7-H3 to tumor cell plasticity, which can be explained based on different aspects, such as the existence of stem cells and circulating tumor cells, EMT, and tumor metabolic heterogeneity. Last but very significant is the immune plasticity, which enables tumor cells to evade immune surveillance.

B7-family proteins can modulate distinct immune responses through co-stimulatory or co-inhibitory signaling, depending on the specific receptors they bind to. While the receptors of other members of the B7 family have been identified, the receptor for B7-H3 remains elusive. Three putative receptors TREM like transcript 2 (TLT-2), interleukin-20 receptor subunit α (IL20RA), and phospholipase A2 receptor 1 (PLA2R1) have been identified, but it is not thoroughly characterized, and more functional validation is required [12].

B7-H3 is a multifunctional protein primarily known for its role in immune surveillance involving T-lymphocyte activation. However, its role as a costimulatory or co-inhibitory molecule in regulating T-cell response to a specific antigen is an area that requires more investigation. In the TME, B7-H3 exhibits a more inhibitory role on immune cells that comprise the tumor niche, favoring tumor cells to evade an immune attack [13]. To facilitate this, B7-H3 mediates various mechanisms that promote evasion of the tumor immune system by inhibiting apoptosis, phenotypic and functional modulation of tumor-infiltrating macrophages, and secretion of pro-inflammatory cytokines by dysfunctional immune cells. Activation or inhibition of T-lymphocyte proliferation and differentiation depends on the costimulatory or co-inhibitory signals involving T-lymphocyte receptors, the major histocompatibility complex (MHC), B7-family proteins, and many other costimulatory molecules. B7-H3 activates T-lymphocytes, interferon (IFN)-γ secretion, enhances cytotoxic T-cell activity, and boosts the proliferation of cluster of differentiation (CD) 4^+^ and CD8^+^ T-cells in pancreatic cancer [9, 14].

Clinically, upregulated B7-H3 expression is positively associated with poor prognosis and patient survival. Quantitative analysis on the expression of B7-H3 in cancer tissues has been significantly associated with the T-stage, lymph node metastasis, and tumor node metastasis (TNM) stage (Fig. 1) [15]. Resistance to therapy is commonly encountered during anticancer treatment, along with changes in the tumor immune landscape and extracellular matrix re-organization preceding therapy resistance. Given the status of a novel immune checkpoint protein and its preferential expression in cancers, B7-H3 is an ideal candidate for targeted therapy. Advancements in immunotherapy research have yielded diverse modalities in targeting immune checkpoint proteins (Fig. 1). This includes usage of monoclonal antibodies alone or with conjugated drug or radioisotope. Other strategies include administering genetically engineered T-cells carrying receptors that can recognize antigen on cancer cells and kill them (Fig. 1).

### B7-H3 and its significance in CRC

Investigation into the expression of B7 family of proteins in colorectal tissue has revealed that B7-H3 is lowly expressed in normal colon tissue compared to CRC tissue. This feature makes B7-H3 a potential candidate biomarker for the early diagnosis of CRC [16]. Recent studies on CRC tissue samples showed a negative correlation between the tumor-infiltrating T-lymphocyte level and B7-H3 expression [17]. The contradictory nature of B7-H3 can be related to unidentified receptors. Studies comparing B7-H3 in the lesions of CRC at various stages, such as polyps (benign), adenomas (pre-cancerous), high-grade neoplasms (adenomas with higher risk of progressing into cancer), and cancerous tissues, reported high levels of B7H1 and B7-H3 in the early stage of CRC development. They continued to express in various stages of CRC, such as adenomas, high-grade neoplasms, and cancer [16]. However, the expression of B7-H4 was reported only in the advanced stage i.e., the CRC.

Interestingly, immunohistochemistry studies revealed differences in their subcellular localization of B7-H1 and B7-H3 depending on the stage of CRC development [16]. Peng Lu and co-workers conducted a bioinformatics study on the genes in CRC cells and normal colon cells that function together with B7-H3, and they found that the gene expression was altered according to the activity of B7-H3. They reported that B7-H3, which functioned at the plasma membrane, brought a critical activation to the lymphocyte, thus validating the findings by other groups on the immunological role of B7-H3 in T-cell response. In-depth studies into the biological pathways revealed that B7-H3 was involved in T-lymphocyte receptor signaling, leading to automatic immune resistance [18]. More details on T-cell regulation by B7-H3 in TME are described in Sect. 2.4. Investigations on B7-H3 are generating considerable interest in cancer research, as it is a promising diagnostic and prognostic marker and a viable target for anticancer treatment [19–21]. Indeed, various clinical studies have well-established the association between B7-H3 and poor prognosis [10]. The development of immunotherapy targeting B7-H3 is rapidly evolving, with many ongoing clinical trials investigating the safety and efficacy of these therapeutic approaches in cancer patients. Details on various strategies targeting B7-H3 are given in Sect. 6. Overall, B7-H3 is emerging as a more promising prognostic and potential candidate for CRC treatment.

### Non-immunomodulatory role of B7-H3 in CRC progression

Apart from the well-studied immunomodulatory action of B7-H3 and its link to CRC, this multifunctional protein contributes significantly to changes in the TME and tumor plasticity, promoting CRC occurrence and progression (Table 1). Tumor plasticity refers to a non-mutational process [22] that is closely associated with processes such as epithelial-mesenchymal transition (EMT), cancer stem cell (CSC) formation, metabolic reprogramming, etc. [23]. Phenotypic plasticity underlies local invasion and distant metastasis in CRC [24, 25]. Furthermore, tumor heterogeneity, senescence, relapse, and treatment resistance are a consequence of tumor plasticity [26].

**Table 1:** Molecular effectors of tumor plasticity and role of B7-H3 in CRC progression

Mechanisms promoting tumor plasticity	Type of study	Effectors of tumor plasticity	Mechanism of action and outcomes	References
Angiogenesis	*Clinical*, CRC tissue samples	VEGF, HIF-1α	Upregulated B7-H3 expression activated VEGFA and CD31 via NF-κB pathway	[27, 28]
	In vitro, HCT116, HCT8, SW480, SW620 and Caco-2	VEGF, HIF-1α	High B7-H3 expression activated VEGFA via AKT1/mTOR signaling	[29, 30]
Apoptosis	*Clinical*, CRC specimens	Bcl-2, Bcl-xL, caspase-8, caspase-9	Low level of apoptosis is associated with high expression of Bcl-xL and Bcl-xL causes upregulation of transcription factor c-MYB, controlling cell cycle	[31]
	In vitro, HCT-116, SW480 cells SW620 and HCT8 cells	Caspase-3, β1-integrin, cyclin D1, FAK, Bcl-2 and Bcl-xL	Upregulated B7-H3 triggered JAK2/STAT3 pathway, promoted Bcl-2 and Bcl-xL expression, and inhibited caspase-3 expression	[32, 33]
CSC	*Clinical*, CRC tissue samples	ALDH1, CD133	High expression of B7-H3 correlated with upregulated CD133 expression High expression of Oct4 correlated with liver metastasis	[2, 34, 35]
	In vitro, HCT-116 cells	NF-κB, MMP9, ALDH1, CD44, CD133	Overexpression of B7-H3 induced Smad1 protein via PI3K/AKT signaling and upregulated Oct4, CD44, CD133 expression	[30, 36, 37]
EMT	In vitro, HCT-116, RKO, SW480, Caco-2 cells	Slug, vimentin, E-cadherin, NF-κB, FAK	Overexpression of B7-H3 was associated with an elevation of MMP2, MMP9, N-cadherin, vimentin and inhibited E-cadherin and β-catenin expression Upregulated B7-H3 promoted JAK2/STAT3 signaling and MMP9 expression	[36, 38, 39]
	*Clinical*, CRC tissue samples	MMP2 and MMP9	B7-H3 activates PI3K-Akt pathway and upregulating the expression of Smad1	[36]
Metabolism	*Clinical*, CRC tissue samples	GLUT1, HK1, LDH, PKM2	High B7-H3 expression influenced FBG, FMN, and LDH levels	[21, 40]
	In vitro, HT-29, Colo-205, HCT-116, RKO cells	PEPCK, lactate	Increased B7-H3 promoted lactate and HK2 production	[41, 42]

*ALDH1* aldehyde dehydrogenase 1, *B7-H3* B7 homolog 3 protein, *Bcl-2* B-cell lymphoma 2, *Bcl-xL* B-cell lymphoma-extra large, *CD* cluster of differentiation, *CRC* colorectal cancer, *EPCAM* epithelial cell adhesion molecule, *FAK* focal adhesion kinase, *FBG* fasting blood glucose, *FMN* fructosamine, *GLUT1* glucose transporter 1, *HIF1* hypoxia-inducible factor 1, *HK* hexokinase, *HK2* hexokinase 2, *JAK* janus kinase, *LDH* lactate dehydrogenase, *MMP* matrix metalloproteinase, *NF-κB* nuclear factor kappa light chain enhancer of activated B-cells, *PEPCK* phosphoenolpyruvate carboxykinase, *PKM* pyruvate dehydrogenase, *STAT* signal transducers and activators of transcription, *VEGF* vascular endothelial growth factor

Tumor plasticity allows tumor cells to evade apoptosis, invade, and metastasize to distant places [32]. B7-H3 has enabled CRC cancer cells (SW620 and HCT8 with varying expression levels of B7-H3) to resist apoptosis and enhance cell survival [32]. This anti-apoptotic property (Table 1) is enabled via activating the Janus kinase (Jak)2/signal transducers and activators of transcription (STAT) 3 pathway that increases the expression and levels of anti-apoptotic proteins B-cell lymphoma 2 (Bcl-2) and B-cell lymphoma extra-large (Bcl-xL) while decreasing levels of pro-apoptotic Bax [32]. Thus, B7-H3 confers resistance to apoptosis induced by anticancer drugs. This observation also applies to pancreatic and gastric carcinoma, where B7-H3 has shown similar anti-apoptotic features [43, 44]. Sun et al. described an alternative mechanism associated with the anti-apoptotic effect of B7-H3. As reported in gastric cancer, B7-H3 interacts with fibronectin, which consequently activates the oncogenic signaling pathway phosphoinositide 3-kinase (PI3K)/protein kinase B (AKT), inhibiting apoptosis [44]. Two downstream targets in PI3K/AKT pathway related to apoptosis (Bcl-2 was upregulated) and cell cycle progression (p53 was downregulated) were dysregulated by the direct interaction of B7-H3 with fibronectin [44].

**Fig. 2:**
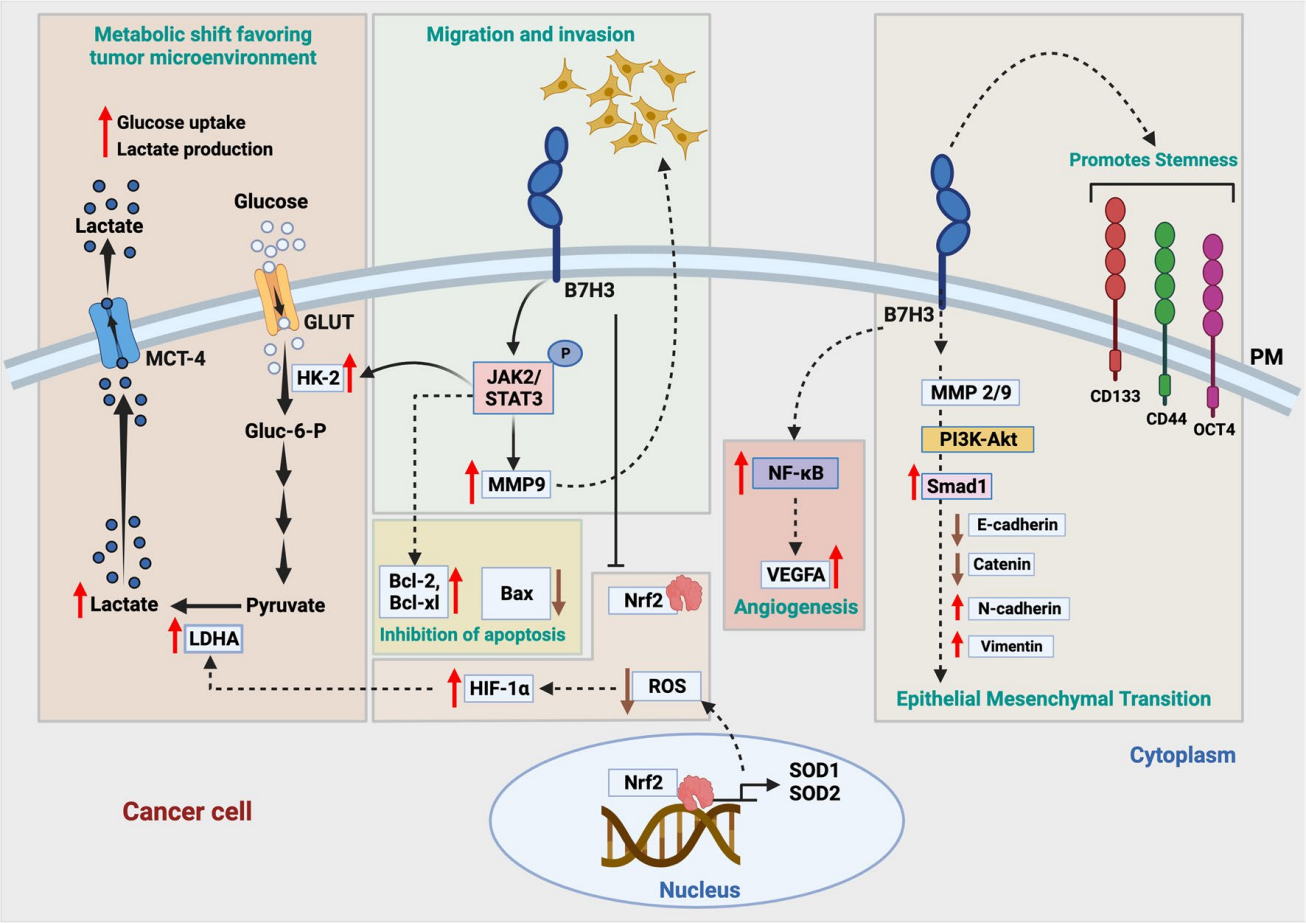
The central role of B7-H3 on factors regulating tumor cell plasticity and tumorigenicity. B7-H3 is highly upregulated in cancer cells. It exerts a pivotal role in cancer progression and is linked to cancer hallmarks such as EMT, angiogenesis, altered tumor metabolism, CSC, inhibition of apoptosis, migration, and invasion. Moreover, B7-H3 also has a prominent role in tumor immune regulation (discussed in Fig. 4). Created with BioRender.com

Another feature of tumor plasticity known as EMT is the reversible functional and phenotypic changes that enable the malignant cells to invade and disseminate to distant sites. Cancer cells with high metastatic potential exhibit increased B7-H3 expression in various cancers, including CRC [45]. Bo Jang and co-workers conducted a study on CRC, emphasizing the significance of B7-H3 in inducing malignant transformation [36]. To evaluate the metastatic potential associated with B7-H3 expression, they looked at the matrix metalloproteinases (MMPs), epithelial markers, and acquired mesenchymal markers in SW480 and Caco-2 CRC cell lines [36]. Epithelial markers β-catenin and E-cadherin were downregulated, while the mesenchymal marker vimentin and N-cadherin were upregulated in B7-H3 overexpression experiments [36]. Closer analysis revealed that Smad1, a transcription factor, is translocated to the nucleus to transcribe the downstream genes regulating EMT and was found to be regulated by B7-H3 via PI3K/AKT signaling [36]. This hypothesis proves that B7-H3 is a key regulator in B7-H3-induced EMT. Moreover, MMP2 and MMP9 were co-expressed in CRC tissues with B7-H3 and clinically correlated with CRC patient’s T-stage [36]. Mechanistically, MMP expression is regulated by the phosphorylation of STAT3 [38]. B7-H3-overexpressing cells activated the Jak2-STAT3 axis pathway, activating downstream MMPs, crucial modulators for cancer cell migration and invasion [38]. In the context of tumor plasticity, stemness is another feature that needs to be discussed along with EMT (Table 1). Cancer cells exhibiting EMT traits have shown characteristics of CSC [23, 46]. The metastatic spread of cancers correlates with the expression of stem cell biomarkers CD133, CD44, ALDH1, and OCT4 [2, 47–50], which was significantly increased in CRC cells overexpressing B7-H3 (Fig. 2). Clinically, the expression of CSC markers in CRC correlated with liver metastasis [2]. An independent study by Zhou Bin and co-workers reported co-expression of CD133 and B7-H3 in 22 of the 104 CRC patient tumor samples [34]. The co-expression of these proteins is clinically correlated with tumor invasion, both lymphatic and distant metastasis, and Duke’s stage, thus proposing B7-H3 as a reliable prognostic marker [34]. CD133 has already been associated with CSC, and its presence is related to tumor initiation and poor prognosis in CRC [51]. Moreover, CD133 also aids in immune evasion. Nevertheless, the mechanism of interactions between the two molecules has not yet been elucidated as their receptors have not yet been identified or characterized [34]. Tumor angiogenesis is a critical step toward tumorigenesis, invasion, and metastasis with a proven role of B7-H3 participation (Table 1). Different components of the TME create a favorable environment for neovascularization mediated via cytokines and other growth factors collectively known as angiogenic factors. The latest findings provide insights into a novel mechanism of angiogenesis by cancer-secreted exosomes [29]. Exosomes are small vesicles containing proteins, DNA, and RNA secreted by different types of cells, including malignant cells, mediating intracellular communications. CRC-derived exosomes with overexpressed B7-H3 were found to be taken up by vascular endothelial cells [29]. This angiogenic program mediated by B7-H3-expressing exosomes was achieved through the AKT1/mTOR/VEGFA signaling pathway [29]. Other significant findings on angiogenesis in CRC demonstrated B7-H3/NF-κB/VEGFA axis in VEGFA expression. While examining the mRNA levels of key angiogenic cytokines VEGFA, VEGFC, bFGF, and PDGF-BB, a positive association was reported with the B7-H3 expression levels [27]. NF-κB activity was more closely involved in promoting angiogenesis than the AKT or STAT3 pathway, as indicated by B7-H3-dependent VEGFA expression [27].

In addition, microRNAs may play an essential role in regulating CRC growth and progression. miR-29a inhibits colon cancer progression, invasion, and migration via downregulation of B7-H3 expression [52]. These data point to miR-29a and B7-H3 as novel molecular targets for advanced CRC chemotherapy. Wang et al. found the suppression of CRC growth, migration, invasion, and cell apoptosis induction through miR-187 overexpression. However, the activation of B7-H3 showed opposite effects on CRC cells. This study documented the essential role of miR-187 in suppressing CRC progression by directly affecting B7-H3 [53]. Besides that, in vitro and in vivo evaluations documented that TGF-β1 supports CRC immune escape by overexpressing B7-H3 and B7-H4 through the miR-155/miR-143 signaling [54]. Another study elucidated a miR-34a-modulated cancer immune evasion mechanism via overexpressed B7-H3 in CRC in vitro and xenograft models. More specifically, miR-34a downregulated SIRT1 and activation in NF-κB/B7-H3/TNF-α signaling pathway [55].

### Association between B7-H3 and tumor glucose metabolism in CRC progression

**Fig. 3:**
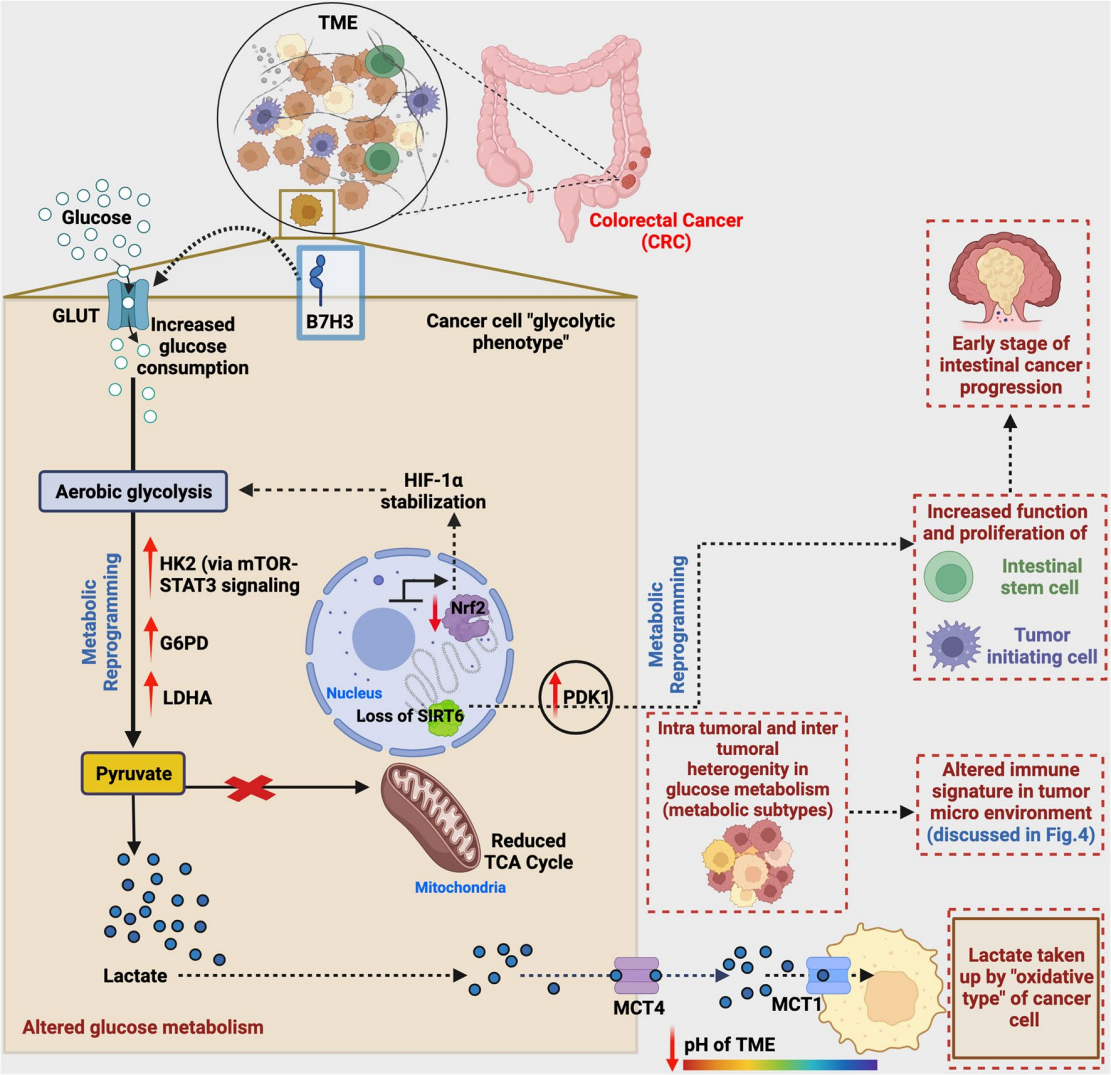
Overview of metabolic reprogramming by B7-H3 in CRC progression. B7-H3 is highly expressed in CRC patients. At the cellular level, it regulates glucose metabolism in CRC cells via increased expression of HK2, G6PD, and LDHA, where HK2 is a downstream target of B7-H3. Additionally, B7-H3 promotes increased uptake of glucose consumption and conversion of pyruvate to lactate through aerobic glycolysis rather than channeling pyruvate to the TCA cycle, leading to lactate accumulation. These results lead to altered pH conditions. Lactate released via MCT can be taken up by oxidative type of tumor cells via the MCT1 transporter, favoring a symbiotic association between different metabolic subtypes. A rare population of quiescent cells expressing intestinal CSC markers with glycolytic phenotype exhibits high PDK activity with enhanced tumorigenic potential. The enhanced PDK1 activity is due to the loss of inhibition by the nuclear factor SIRT6 over PDK1 expression. This metabolic reprogramming causes increased proliferation of tumor-initiating cells progressing to an early stage of CRC. As the metabolism is heterogeneous among the population of CRC cells, it will lead to the generation of variant metabolic subtypes, enabling the progression of CRC. Information in the red dotted box represents the outcome of metabolic reprogramming and the after-effects. Created with BioRender.com

The development of CRC is a progressive process with an accumulation of changes at the genetic, epigenetic, cellular, morphological, molecular, and metabolic levels. Altered cellular metabolism, now considered a hallmark in CRC progression, is known to modify the landscape of the tumor environment. The abnormal metabolic program provides tumors with alternative energy resources supporting the continuous existence of CRC cells. Glucose, the primary energy source, is catabolized in a normal cell through multiple steps via the glycolysis-oxidative decarboxylation-Krebs cycle-oxidative phosphorylation mechanism that occurs in the presence of oxygen. Alternatively, in the absence of oxygen, pyruvate is converted to lactate. Otto Warburg was the pioneer to report metabolically different phenotypes in cancer where, even in the presence of oxygen, he observed a shift in glucose metabolism towards glycolysis with increased lactate production and reduced oxidative phosphorylation (this mechanism is famously known as the “Warburg effect”). This shift in tumor metabolism can significantly influence the TME by remodeling the immune and neoplastic cells that comprise the TME [5]. B7-H3 compliment CRC progression by regulating glucose metabolism (Fig. 3 and Table 1), and the abnormal level of metabolites secreted influence the type and function of the immune cells in the TME, further promoting chemoresistance. B7-H3 has been documented as a critical immune-independent contributor to cancer glucose metabolism reprogramming via ROS-mediated stabilization of HIF-1α [56].

Shi et al. illustrated the direct link of B7-H3 in promoting aerobic glycolysis by regulating one of the key enzymes hexokinase type 2 (HK2) [42]. The study reported elevated B7-H3 expression followed by a significant increase in glucose consumption and lactate production, while depletion of B7-H3 exhibited the opposite effects in HCT-116 and RKO cells [42]. They further explored the molecular pathway by which B7-H3 mediates the HK2 expression, where they found that STAT3 signaling regulates the expression of HK2 and STAT3 as a downstream target of B7-H3 in CRC cells (Fig. 3) [42]. Shi et al. also verified a positive correlation between the expression of B7-H3 and HK2 in tissue samples from CRC patients, thus further emphasizing the role of B7-H3 in CRC cancer progression [42]. Consistent with this observation, a positive correlation between tumor glycolysis and immune signature in TME was recorded across 14 cancers [57]. A computational analysis of glycolysis-related genes (e.g., hexokinase and phosphoglucose isomerase in the glycolytic pathway) was upregulated in highly glycolytic cancers [57].

### Immunomodulatory role of B7-H3 in relation to tumor immune evasion/surveillance and CRC progression

The role of B7-H3 in the TME and its immunoregulatory function is an active area of research. TME is a unique ecosystem that emerges due to interaction with several host cell components. Additionally, the TME is a continually evolving system composed of neoplastic cancer cells and multiple stromal cells, including angiogenic endothelial cells, pericytes, infiltrating immune cells, and cancer-associated fibroblasts (CAFs) releasing mitogenic factors capable of promoting cancer cell proliferation [58]. Tumor immune evasion and metastatic tendency in CRC are achieved through intrinsic plasticity and adaptability orchestrated by cancer cells, immune cells, and stromal cells via cytokines, growth factors, and other signaling molecules [37, 59, 60]. Cancer cells can invoke an immune response that could either drive a cytotoxic immune reaction or an antagonistic response with a proliferative effect. The innate and adaptive immune systems participate in the immune recognition and elimination of neoplastic cells. An innate immune system comprising macrophages, natural killer cells, neutrophil granulocytes, and monocytes is the first line of defense against invading pathogens and drives the host recognition of cancer-associated antigens [61]. Host recognition of cancer cells initiates active recruitment of T-lymphocytes into the tumor area, and other immune cells of the innate immune system, along with T-lymphocytes, orchestrate the antitumor action. However, cancer cells escape immune surveillance through immune evasion. As discussed in the previous chapter, metabolic reprogramming in the tumor niche prevents the tumor cells from being recognized by the immune system. Cancer cells employ various cell survival signaling pathways to escape antigen recognition by the release of immunosuppressive mediators, creating an acidic environment, recruiting tumor-promoting immune cells (macrophages with an M2-phenotype), expansion of tumor-promoting T-lymphocyte signature under the influence of chemokine and cytokine secreted by tumor cells [59]. The human Vγ9Vδ2 T cells represent a specific cell type that shows great potential in immunotherapy cancer research. In this regard, the suppression of the B7-H3 activity significantly elevated the IFN-γ-dependent cytotoxicity of Vγ9Vδ2-T cells against colon cancer cells in the mouse HCT116 xenograft model [62].

**Fig. 4:**
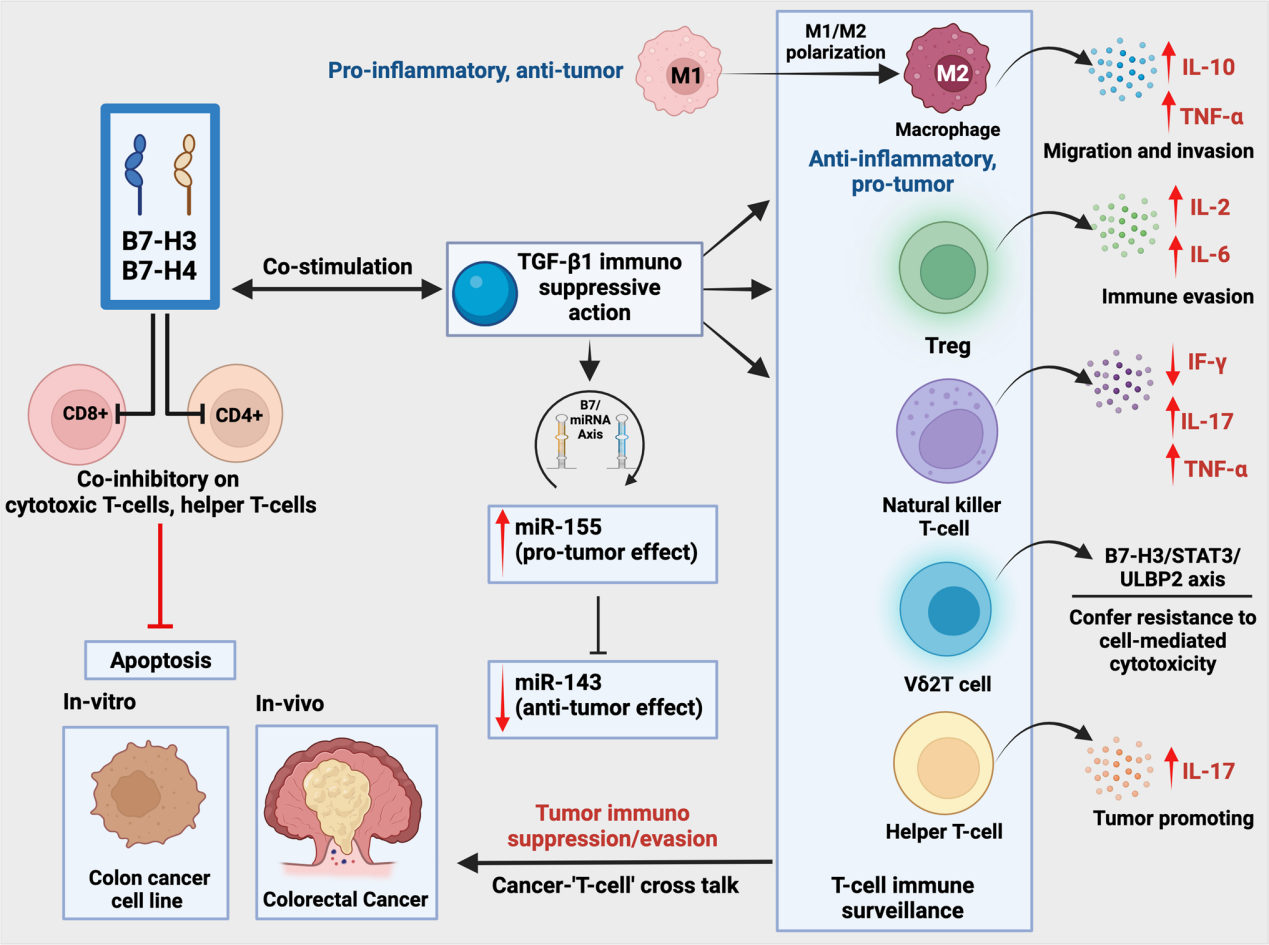
Role of B7-H3 on the plasticity of immune cells in shaping TME. B7-H3 are abnormally high in cancer cells and are inducible in some immune cells like antigen-presenting cells (APCs) and macrophages. TAM (tumor-associated macrophage) is the predominantly occurring immune cell in TME, and B7-H3 receptors are highly expressed on TAM. B7-H3 in the cancer cells drives the phenotypic transformation of M1 to M2 macrophage in the TME, favoring altered immune function and suppression. The autocrine action of TGF-β1 increased the expression of miR155 while downregulating the expression of miR143 in CRC and T-lymphocytes exposed to TGF-β loses their capability to kill neoplastic cells [54]. Also, TGF-β1 increased the proliferation of immunosuppressive Treg cells. The immunomodulatory action of B7-H3 limits the proliferation and function of CD4+ and CD8+ T-lymphocytes through the inhibition of nuclear factor kappa B (NF-κB). Furthermore, B7-H3 inhibits NK cell antitumor activity. Vδ2T, a subset of T-lymphocytes with increased cancer cell killing capacity [68], is attenuated by enhanced expression of B7-H3 molecule expressed on cancer cells via a molecular pathway involving Signal transducer and activator of transcription 3/UL16 binding protein 2 (STAT3/ULBP2) signaling [69]. B7-H3-induced cytokine (IL-2, IL-6, IL-17) secretion by T-lymphocytes generates a conducive niche for immune suppression /immune evasion favoring CRC progression. Created with BioRender.com

Histopathological analysis of CRC tissue showed strong evidence of tumor-infiltrating lymphocytes (TILs) and tumor-associated macrophages (TAMs) in tumor tissue, indicating their role in adaptive and innate immunity. TAMs are seen predominantly in two functional phenotypes, M1 and M2 (Fig. 4). The former is anti-tumorigenic. It induces both direct cytotoxic effect and antibody-dependent cell-mediated cytotoxicity, while the latter is pro-tumorigenic and pro-angiogenic with the expression of interleukin (IL)-10, IL-1β, VEGF, and MMP [63]. M1 and M2 phenotypes have high plasticity depending on the TME [63, 64]. An increased presence of tumor-infiltrating macrophages has been associated with cancer cells with more B7-H3. Clinically, a high expression of B7-H3 and the TAM density in CRC tissue were associated with reduced overall survival of patients [65]. Studies examining the immune cells infiltrating the TME revealed that the M2 phenotype was predominantly present in the TME and indicated the presence of pro-tumor growth factors and cytokines released by M2 macrophage [10, 65]. The JAK2-STAT3 pathway could probably mediate the polarization of macrophages [66]. Closer analysis showed the presence of CD68^+^ macrophages in both tumor nest and stroma, and it positively correlated with B7-H3 expression on cancer cells. Concerning T-lymphocyte infiltration, flow cytometry analysis for the expression of B7-H3 on CD3^+^ T-lymphocytes showed significant differences with the adjacent normal tissue [16]. As the receptor for B7-H3 is not characterized yet, explaining the mechanism of its immunoregulatory role is challenging. However, putative receptors for B7-H3 have been identified on activated T-lymphocytes and TAMs [65]. A study by Mielcarska et al. provides an exemplary observation of B7-H3 expression in the context of the immune landscape in CRC [10] investigating the CRC tissue’s tumor status, TIL, and cytokine composition [10]. The immune infiltration cell analysis showed a clear shift in the density of immune cells in the tumor correlated with B7-H3 expression [10]. Tumors with elevated B7-H3 expression showed reduced levels of CD4^+^ quiescent memory T-lymphocytes and CD4^+^ activated memory T-lymphocytes [10].

On the other hand, increased levels of regulatory T-cells (Tregs), macrophages M0, monocytes, and neutrophils were associated with tumors with increased B7-H3 expression [10]. Increased IFN-γ and tumor growth factor (TGF)-β responses were also elevated [10]. Additionally, the shift in the macrophage polarization from M1 to M2, as indicated by the CD163 marker, further confirmed the immunoregulatory role of B7-H3 in shaping TME. To determine the functional role of B7-H3 in cell signaling, Mielcarska et al. performed gene set enrichment analysis (GSEA). The study revealed the upregulation of critical metabolic pathways corresponding to oxidative phosphorylation, fatty acid metabolism, and heme metabolism [10]. On the contrary, high expression of B7-H3 showed downregulation of metabolic pathways associated with hypoxia, glycolysis, mTOR C1 signaling, and G2M checkpoint [10]. Nevertheless, the observation of the downregulation of hypoxia and glycolysis-related pathways in this study conflicts with the observations by other research groups [42, 67].

## Association between gut microbiota and B7-H3 in CRC

The recent resurgence in microbiome research has revolutionized advances in precision and preventive medicine, diagnostics, and health and wellness. Microbial signatures associated with specific diseases can validate the diagnostics and therapeutic intervention, leading to a more precise outcome. Various research groups have studied the microbiome and its relation to CRC. Among the studies, fecal metagenomics data collected from 120 samples from CRC patients and tumor-free controls indicated the presence of a reproducible taxonomic signature associated with CRC type [70]. Interestingly, an article published by Peuker et al. described a microbiota-dependent crosstalk between B7-H3, myeloid cells, and T-lymphocytes, suppressing CD8^+^ T-lymphocytes-dependent antitumor immunity [24]. The underlying mechanism involved the local microbial signal within the TME sensed by myeloid cells promoting the activation of immunoregulatory cell signaling via the calcineurin-nuclear factor of activated T-lymphocyte (NFAT) axis. This contributed to IL-6 release, thereby enhancing the expression of B7-H3 and B7-H4 in tumor cells, ultimately culminating in the inhibition of CD8^+^ T-lymphocytes-dependent antitumor immunity. Compromising the integrity of the intestinal barrier is crucial in initiating inflammation and progression of CRC. The study by Peuker et al. [71] was primarily conducted in mice and found that intestinal epithelial cells constitutively expressed NFAT and calcineurin. They undergo selective activation in tumor tissue by impaired stratification of the tumor-associated microbiota and toll-like receptor (TLR) signaling. Microbiota-dependent activation of the calcineurin-NFAT axis promotes CSC proliferation and suppresses apoptosis [71]. Hence, gut dysbiosis promotes cancer by suppressing the antitumor immune response. The latest paper by Jaensch et al. provides a comprehensive overview of the role of microbiota in CRC from a clinical perspective, giving insights into the basic and translational research [72].

## Clinical correlation of B7-H3 in CRC

Disrupted glucose metabolism, a distinctive feature of diabetes mellitus (DM), is a biochemical hallmark of cancers, including CRC. Patients with DM exhibit a higher susceptibility (27%) to CRC than individuals without diabetes [73]. High levels of three clinical indicators, such as fasting blood glucose (FBG), a significant criterion for DM, lactate dehydrogenase (LDH), a key enzyme of glycolysis, and flavin mononucleotide (FMN) indicating the precise blood glucose concentration were identified as independent predictors of poor overall survival (OS) in CRC patients. Clinically, as shown in Table 2, B7-H3 positively correlated with tumor invasion, lymph node metastasis, and tumor node metastasis (TNM) stage [74]. As reported by Ingebrigtsen, V. A et al., for FBG levels, 32.9% of the cases exhibited high levels of expression and positive rates of 35.7% for LDH and 21.1% for FMN (cutoff value of 169.5 and 202.5, respectively) with significant correlation to tumor location, lymph node metastasis, and TNM.

**Table 2:** The influence of B7-H3 in CRC—clinical studies

No. of patients; type of sample/experiment	Key findings	Year	Reference
102 CRC patients; pathologic specimens	B7-H3 level was increased in serum and tissue from CRC patients	2010	[17]
277 CRC patients; paraffin-embedded samples	B7-H3 was overexpressed in the cell membrane, cytoplasm, and nucleus of CRC cells. B7-H3 was associated with metastasis and reduced OS	2012	[74]
731 CRC patients; tissue microarrays	B7-H3 was upregulated in CRC cells	2014	[75]
104 CRC patients; tumor samples	B7-H3 was highly expressed and correlated to CRC progression, metastasis, invasion, and poor survival	2014	[34]
1202 CRC patients; meta-analysis	B7-H3 expression correlated significantly with metastasis progression and a reduced OS	2016	[76]
213 CRC patients; paraffin-embedded samples	B7-H3 was overexpressed in CRC cells and associated with metastasis as well as poor prognosis	2020	[21]
125 CRC patients; tumor samples	B7-H3 promoted CRC angiogenesis, invasion, metastasis and was found in abundance	2020	[27]
805 CRC patients; tissue microarrays	B7-H3 was upregulated and associated with CRC aggressiveness and a poor prognosis	2020	[68]
214 CRC patients; tissue microarrays	Low B7-H3 and PD-L1 correlated with a significantly better OS	2021	[77]
90 CRC patients; preoperative serum analysis	B7-H3 was found to be higher; the higher the stage or, the more advanced the metastasis was	2022	[19]

*B7-H3* B7 homolog 3 protein, *CRC* colorectal cancer, *OS* overall survival, *PD-L1* programmed death-1 ligand

In addition, a linear correlation was observed between B7-H3 and FBG with tumor invasion, lymph node metastasis, and TNM in the early stage of CRC. A worse OS was observed in a subgroup with high B7-H3 and FMN expression than in a subgroup with high FBG and LDH [21]. Hence, B7-H3 combined with FBG, LDH, and FMN could guide the clinician with prognosis. Clinical studies showed high expression of B7-H3 in CRC tissue with a positive rate of 63.8% out of 213 patients recruited for this study [21]. Immunohistochemical studies of CRC tissue samples revealed B7-H3 predominantly localized in the cytoplasm and on the plasma membrane of the cancer cells and is significantly higher in tumor tissue than in normal colorectal tissue. Similar studies published by another group also indicated the expression of B7-H3 in the cell membrane and cytoplasm of the CRC tissue samples by 62% and 46%, respectively (Table 2). This study (and others) also reported a link between nuclear localization and poor prognosis with vascular invasion in CRC patients [34, 74]. More studies on the cellular localization of B7-H3 in CRC tissue indicated a tumor stage-dependent expression of this co-inhibitory protein. For example, during the adenoma stage, B7H1 and B7-H3 were primarily expressed in the nucleus, along with their expression in tumor-infiltrating lymphocytes.

In contrast, they were more expressed in the cytoplasm and membrane in the high-grade neoplasia stage. Interestingly, B7-H4 was only expressed in the colorectal carcinoma stage, and furthermore, the higher expression of B7-H3 in colorectal lymphocytes correlated with lymph node metastasis. Overall, based on the 7-year survival analysis by Zhichao et al., patients with high expression of B7-H1, B7-H3, and B7-H4 of the B7 family had a worse prognosis than patients with low expression [16].

However, later studies by Zhichao et al. could not prove the prognostic relevance of nuclear B7-H3(nB7-H3), which might have been due to the differences in the methodology used, i.e., whole tissue sections (WTSs) vs. single core tissue microarray (TMA) sections. Nevertheless, regarding nB7-H3 association with patient outcome, there was a significant correlation between nB7-H3 and reduced disease-free survival in TNM stage1 patients [20]. Comprehensive studies on the levels of sB7-H3 (soluble or circulating form) showed elevated levels in the sera of CRC patients [19]. Supporting this observation, in vitro studies using CRC cell lines to understand the underlying mechanism revealed the involvement of inflammatory cytokine TNFα in the increased stimulation of serum B7-H3 (sB7-H3) and the involvement of MMP in the shedding of membranous proteins [17]. The presence of sB7-H3 has been extensively studied in other cancers, such as breast, bone, liver, and pancreas cancers [78–81]. All these studies had a common observation emphasizing the increased serum levels of B7-H3 correlated with poor prognosis in various cancers.

Further, more clinical studies (Table 2) supported the role of B7-H3 as an immunosuppressive protein, as data confirmed a negative correlation with tumor-infiltrating lymphocyte intensity and a positive correlation with tumor grade in CRC patients. The expression of B7-H3 in primary tumors significantly correlated with their metastatic sites and corresponded to an advanced overall stage, poor prognosis, and CD45RO T-lymphocytes infiltration [68]. The membrane and subcellular localization of B7-H3 may determine the functional role of this protein in oncogenesis. The heterogeneous expression of B7-H3 in tumor tissue and its clinicopathological relevance to patient outcome warrants further investigation. Apart from B7-H3’s immune evasion and pro-proliferative action, it also reprograms the tumor vasculature. Based on the microvascular density (MVD) study performed in 125 CRC patients, B7-H3 promotes tumor angiogenesis. Immunohistochemistry staining with CD31, a specific endothelial marker for validating MVD demonstrated a positive correlation with the expression of B7-H3 in CRC tissue, which increased with lymph node metastasis and advanced stage of cancer (stage III and IV) [27].

Furthermore, a subset of CRC cells exhibiting CSC characteristics, with CD133, was found to co-express with B7-H3 in CRC tissue. CD133 is another crucial biomarker associated with CSC [34, 48]. Its presence is a marker for poor prognosis and negatively correlates with survival in CRC patients. Notably, the CRC group positive for both B7-H3 and CD133 exhibited high lymph node metastasis, distant metastasis, and enhanced tumor invasion. Indeed, the presence of B7-H3 on CRC stem cells helps to evade immune surveillance and demonstrates a link with the pathological status of CRC. Some groups analyzed the transcripts of this protein in peripheral blood mononuclear cells (PBMCs) in patients with adenomatous polyps and healthy individuals and demonstrated a link with the pathological status of CRC. They showed a significantly high level of peripheral blood transcripts (PBTs), which correlated with tumor invasion and the advanced stage of TNM [82]. Overall, across various studies (Table 2), the percentage of B7-H3-positive CRC tissue varied between 50–80% of the cases [20, 76]. A study in a multi-racial patient population with metastatic CRC, with low PD-L1 and B7-H3 expression, was associated with an improved prognosis [77]. The expression of PD-L1 and B7-H3 protein was independent of racial disparities.

Since obesity is considered a risk factor for CRC, the B7-H3-driven metabolism of adipocyte progenitor cells was investigated in relation to the development of obesity in mouse models [83]. Nevertheless, the connecting link between obesity, B7-H3, and CRC risk has not yet been explored.

## Role of B7-H3 in CRC chemoresistance

Resistance to chemotherapy is an acquired or adaptive trait developed by cancer cells to escape the cytotoxic effects of chemotherapeutics. The resistance acquired due to altered drug metabolism, drug transport, and epigenetic factors can be attributed to tumor cell plasticity [22, 84]. Exclusively, TME has a significant role in facilitating chemoresistance [30, 85]. The role of B7-H3 in EMT was discussed earlier. As TME promotes EMT transition [86], mesenchymal tumor cells cause the upregulation of TGF-β signaling and generate a positive feedback loop on EMT. Concurrently, this promotes the generation of Forkhead box P3 + (Foxp3) Treg cells, which antagonizes the activity of cytotoxic T-cell and NK cells, resulting in immune therapy resistance and chemoresistance. Accumulating evidence emphasizes altered glucose metabolism in CRC progression and therapy resistance. Earlier, we discussed the interdependent role of glycolysis and B7-H3 in CRC progression and apart from that B7-H3 also promotes chemoresistance [42]. An interesting paper by Tongguo Shi et al. in 2019 discusses the role of B7-H3 in aerobic glycolysis and chemoresistance [42]. They showed increased aerobic glycolysis, clonogenic potential, and decreased apoptosis with the B7-H3 overexpression assay.

Furthermore, the effect was reversed by the knockdown of B7-H3. In both in vitro and in vivo studies, they were able to abolish B7-H3-induced L-OHP (oxaliplatin) and 5-FU resistance in CRC cell lines HCT-116 and RKO either by knockdown of HK2 expression or by 2-DG, a glycolytic inhibitor. In 2017, Zhang et al. reported B7-H3-dependent oxaliplatin resistance in CRC cells [87]. Here, they identified another protein upregulated by B7-H3, the X-ray repair cross-complementing group (XRCC1). This protein is related to DNA damage repair, and its upregulation confers resistance to oxaliplatin treatment via the PI3K-AKT pathway. In other words, B7-H3 decreases oxaliplatin-induced DNA damage by enhancing the expression of XRCC1. Another mechanism conferring resistance to chemotherapy was cell cycle-dependent. Drugs that can arrest G2/M-phase can induce apoptosis in cancer cells. However, cells over-expressing B7-H3 have a dramatically lower number of cells in G2/M-phase than the control group after oxaliplatin treatment in CRC cell lines [88]. The mechanism involved the B7-H3 dependent regulation of a protein, called cell division cycle 25A (CDC25A). CDC25A, a cell cycle-regulating protein, is positively correlated with B7-H3 expression and is upregulated in many cancers. STAT3, which is involved in aerobic glycolysis, is also involved in the transcription of CDC25A [88]. Hence STAT3/CDC25A pathway confers resistance in B7-H3 overexpressed CRC cells. In another study, B7-H3 knockdown strongly induced CRC cells’ growth arrest and senescence after DOX therapy.

On the other hand, overexpressed B7-H3 showed the opposite results in the same cells. Besides that, the same results were confirmed in vivo. Authors described that B7-H3 suppressed growth arrest and cellular senescence in CRC via the activation of AKT/TM4SF1/SIRT1 signaling. These data provide a new clinical approach during the chemotherapy of CRC [89].

Radiotherapy is a standard treatment modality for treating CRC. However, it often faces treatment failure, resulting in recurrence and resistance to irradiation. In vitro, B7-H3 mRNA and protein levels were increased in CRC cells after X-ray irradiation. Knockdown of B7-H3 inhibited the radio resistance, and overexpression of B7-H3 resulted in enhanced radio resistance, thus indicating a crucial role of B7-H3 in radiotherapy resistance. In vivo, antibodies targeting B7-H3 sensitized CRC cells to radiotherapy in xenografts in nude mice. In this study, another protein, kinesin family member 15 (KIF15), related to cell cycle progression was positively associated with B7-H3 expression. The activation of KIF15 is regulated through the B7-H3-NF-κB axis, which is modulated via ERK1/2-pathway. Thus, this mechanism protects the CRC cells from radiation treatment by activating the ERK1/2 signaling pathway. Moreover, based on the above observations, B7-H3-expression can be used as a biomarker to evaluate response to radiotherapy [90].

Studies conducted in other types of cancers have shown that silencing of B7-H3 increased sensitivity to rituximab and bendamustine [91]. Resistance to AKT/mTOR inhibitors, triciribidine, and everolimus occurred in B7-H3 overexpressed breast cancer cell lines, and knockdown of B7-H3 reversed the resistance to these drugs via decreasing the glycolytic capacity [67]. Evidence collected from various findings provides a strong association of B7-H3-mediated altered glucose metabolism and chemoresistance. To further understand the metabolic link between B7-H3 and resistance, studying the downstream molecules in the metabolic pathway targeted by B7-H3 is imperative. As mentioned earlier, STAT3 signaling is one such molecule identified, which is a master regulator of glycolysis [92]. All these findings give new insights into the complexity of B7-H3 regarding the multiple functional roles in tumor promotion, resistance, and treatment.

## Targeting B7-H3 in CRC

Based on the impressive outcome from many clinical findings, B7-H3 remains an attractive target for antibody-based therapy as it is heavily present in malignant and cancer stem cells and lowly in normal tissue. Both its immunogenic and non-immunogenic role contributing to pro-tumorigenic function is well documented. Hence, targeting B7-H3 blocks CRC tumor progression, metastasis, and tumor angiogenesis via modulating tumor plasticity [93].

Immunotherapy targeting B7-H3 includes different modalities such as (I) monoclonal antibodies against B7-H3 [62], (II) antigen drug conjugates (ADC), (III) bi- and tri-specific antibodies [94], (IV) chimeric antigen receptor (CAR) T cell therapy, (V) CAR-modified NK therapy, (VI) antibody-dependent cellular cytotoxicity (ADCC) [93, 95, 96], and (VII) dual-affinity re-targeting (DART) recognizes B7-H3 and redirecting T-cells to kill B7-H3 expressing tumors via CD3 (ClinicalTrials.gov NCT02628535). Enoblituzumab, a type of ADCC successfully tested in prostate cancer, can block B7-H3 mediated immune suppression and eliminate cancer cells by triggering antibody-dependent cellular cytotoxicity via NK cells [97]. Apart from immunotherapy, small molecule inhibitors attenuating B7-H3 have been investigated. A recent publication by Wang et al. introduces FDW028, a small molecule inhibitor blocking FUT8, which promotes lysosomal mediated degradation of B7-H3 via the chaperone-mediated autophagy (CMA) pathway [98]. Two recent independent reviews by Kontos and Zhao enlist all the clinical trials targeting B7-H3 in various cancers [93]. Based on the search in clinical trials.gov, there are only two clinical studies about trials in CRC. One of these studies is a TAA06–CART-cell-based clinical trial (clinical trials.gov NCT05190185) using genetically modified T-lymphocytes that express a CAR targeting B7-H3 with immunostimulatory and antitumor activity, ultimately inducing selective toxicity to B7-H3 expressing tumor cells. These clinical trials are still in the recruiting phase; hence, the outcomes are unavailable.

## Discussion

The first report linking B7-H3 to cancer was published in the early 2000s. It was first identified by Chapoval et al. in 2001 as a novel member of the B7 family [9]. Since its discovery, B7-H3 has attracted considerable attention in cancer research due to its potential role in tumor progression and immune regulation. Abnormal expression of B7-H3 has been reported in cancers of the breast, pancreas, prostate, ovary, and brain [78, 99–102]. Its presence in various cancers has been associated with immune evasion and resistance to immune therapy, contributing to a more aggressive phenotype and poor prognosis. For example, several immune checkpoint factors, including B7-H3 have been identified as contributing to chemoresistance, notably in breast cancers [103]. Hence, more studies could yield novel information in improving current anticancer treatment strategies.

Scientists are exploring various aspects of this protein concerning its expression in various types of cancer, stages of cancer, intricate networking with other molecules, and its immune regulatory role in TME, its prognostic and diagnostic significance. Initially, the research on B7-H3 focused on its immune regulatory role, especially in tumor immunity; however, later studies revealed a role going beyond its immunoregulatory function. These emerging studies shed new insights into the functional role and its implications in various physiological and pathological conditions. Nevertheless, its functional complexity is not yet fully understood. In malignant transformation, B7-H3 is implicated in different phases of tumorigenesis, such as tumor progression, inhibition of apoptosis, angiogenesis, EMT, and metastasis. From the papers reviewed B7-H3 strikingly plays a central role in promoting most of the cancer hallmarks. [78, 99–102, 104]

Moreover, its functional role in TME reprogramming has gathered considerable interest from the context of its link with tumor glucose metabolism and immune evasion. Cellular metabolism beyond its scope as an essential requirement for energy and growth of the cell is also associated with the proliferation and metastasis of cancer cells. Emerging studies show that metabolic reprogramming as a cancer hallmark is closely linked to the type of cells comprising stromal cells, immune cells, and gut microbiota. Studies linking tumor metabolism and immunity provide more insight into targeting CRC with a combination of anti-metabolites and immune therapy. An extensive review by Varghese. E et al. link tumor metabolism to anticancer therapy efficacy in breast cancer [105]. Moreover, metabolic subtyping of cancer facilitates a more accurate therapy selection and guide through personalized prognosis management [106]. Combinatory treatment using anti-angiogenic, glucose-lowering agents, immune checkpoint inhibitors, and anticancer agents has yielded promising results [107].

Tumor plasticity or tumor adaptability can have severe implications for treatment response and treatment outcomes, and emerging studies show that B7-H3 can be attributed to tumor plasticity [59]. Since it has a multifaced role in CRC progression, targeting this molecule can disrupt tumor plasticity and ultimately improve treatment response; thus, B7-H3 can be an ideal candidate for targeted therapy. Tumor heterogeneity and tumor plasticity are functionally related terms, and TME is a platform that orchestrates tumor heterogeneity and plasticity. Hence, profiling tumors in terms of metabolic heterogeneity and mapping immune fingerprints of the tumor landscape can guide the selection of therapies that target specific subpopulations or pathways driving resistance.

There are conflicting findings regarding the role of B7-H3 in regulating the immune response. The controversy stems from the observation that B7-H3 can activate or inhibit the T-lymphocyte response in the TME. There are reports on B7-H3 as a costimulatory molecule or a co-inhibitor molecule of T-cell response. After reviewing various findings, it is evident that the effect of B7-H3 on T-cell response depends on factors such as the type of cancer, the experiment model, the expression pattern, expression levels, and the type of cells that make up the TME. Filippos [93] suggests that B7-H3 inhibits adaptive immunity in non-malignant tissue by suppressing T-cell activation and proliferation. However, in malignant tissue, B7-H3 suppresses tumor antigen-specific immune response, which is pro-tumorigenic. Its non-immunologic functions also add to its pro-tumorigenic capacity, which makes B7-H3 an ideal target for cancer treatment. The presence and level of expression of co-inhibitory proteins of the B7 family, especially B7-H3, is clinically relevant in judging prognosis and further guiding the postoperative treatment. Results based on meta-analysis reports mention B7-H3 as a negative predictor of OS and progression-free survival (PFS) in patients with solid tumors [108]. The newly identified soluble or circulating B7-H3 is increased in cancer patients, indicating its potential as a serum biomarker [17]. To further validate sB7-H3 as a diagnostic marker, a large-scale, diverse patient population study is required to test its reliability and sensitivity as a biomarker [80]. Other biomarkers evaluated as biomarkers of CRC include serum Colon Cancer-Specific Antigen-2 (CCSA-2), carcinoembryonic antigen (CEA), and carbohydrate antigen 19–9 (CA19-9) [109, 110]. Though CCSA-2 is a sensitive marker in CRC, it did not correlate with the tumor grade or stage but correlated positively with tumorigenesis. However, CCSA-2 is a good candidate for evaluating surgical thoroughness as their levels decrease significantly after surgery. CCSA-2 occurrence post-surgery during the follow-up period indicates recurrence and poor prognosis. This candidate was found more sensitive as a diagnostic marker than CA19-9 and CEA. Though these markers, including B7-H3, are a potential candidate for prognosis, none of them are good for screening asymptomatic population studies. It is suggested that using a panel of biomarkers for diagnosis and prognosis of CRC enables more accurate understanding of CRC disease progression.

Research on gut microbiota suggests that the composition and function of the gut microbiota can influence CRC development, progression, and anticancer treatment response. Interestingly, specific microbial signatures can contribute to disease progression and influence therapy efficacy. Certain bacteria can influence the antitumor immune response and have been implicated in modulating the response to immunotherapy using immune checkpoint inhibition. Hence, combinatorial therapy using broad-spectrum antibiotics to destroy harmful bacteria can positively aid CRC treatment. It is commendable to note that broad-spectrum antibiotic treatment has decreased tumor size and the extent of liver metastasis in CRC [24]. Given that the existing biomarkers are not particularly effective for population screening, understanding the bacterial signatures and their altered patterns could potentially aid in detecting the early stages of CRC in the asymptomatic population. However, the technology for screening CRC based on gut microbiota has not yet advanced.

Recent advancements in molecular biology and antibody engineering have facilitated the development of strategies targeting B7-H3 utilizing multiple effector mechanisms. These approaches have been successfully tested in vitro and in vivo models. Blocking of B7-H3 (3E8, a specific B7-H3 blocking antibody) combined with irradiation has significantly reduced the tumor size in in vivo tumor models [90]. These encouraging findings provide a foundation for advancing B7-H3-targeting therapies into clinical trials. Noval therapies like ADC, potentially delivering a cytotoxic payload with limited off-target toxicity, have yielded promising results in small-cell lung cancer, osteosarcoma, and glioblastoma [111]. Compared to clinical trials on other cancers, trials on B7-H3 immune checkpoint in CRC are in the early stages. Well-studied immunotherapy using PD-1/PD-L1 inhibitors has shown to be less effective in CRC patients with microsatellite stability (MSS) or low levels of microsatellite instability (MSI-L)[112]. Hence, more reliable immune checkpoint inhibitors are recommended for more efficient treatment of CRC.

A promising immunotherapy approach utilizing genetically engineered T-lymphocytes is known as B7-H3 CAR T-lymphocytes. CAR T-lymphocytes targeting B7-H3 were found to reduce the tumor size in pancreatic ductal adenocarcinoma, ovarian cancer, and neuroblastoma without evident toxicity [113]. The first phase 1 clinical trial of systemic B7-H3 CAR T-lymphocytes in relapsed/refractory solid tumors was safe and demonstrated antitumor activity. To achieve maximum effect, the CAR T-lymphocytes must expand and persist in circulation [114]. The latest technology targeting B7-H3 developed is a tri-specific killer engager (TriKETM) consisting of a nanobody anti-CD16, IL-15, and nanobody anti-B7-H3 held together by a flexible arm [114]. Combined with off-the-shelf NK cells, this construct targets malignant cells expressing B7-H3 by engaging the NK cell’s natural cytotoxicity. This technique is an appealing therapeutic strategy for treating solid tumors with minimum toxicity to normal tissue. Finally, research data shows that the activity of B7-H3 can also be modulated at the mRNA splicing level. Splicing enzyme SRSF3 that directly binds to exons 4 and/or 6 of B7-H3 mRNA may provide another regulatory mechanism for B7-H3 activity during CRC growth and progression.

Given the mounting amount of interest gathered in this field of research, together with its therapeutic implications, B7-H3 holds promise for advancing novel therapeutic strategies for CRC treatment. Therefore, B7-H3 intercedes between tumor plasticity and CRC progression, emerging as a potential therapeutic intervention candidate.

## Abbreviations

ADC: Antigen drug conjugate
ADCC: Antibody-dependent cellular cytotoxicity
AKT: Protein kinase B
ALDH1: Aldehyde dehydrogenase 1
B7-H3: B7 homolog 3 protein
Bcl-2: B-cell lymphoma 2
Bcl-xL: B-cell lymphoma-extra large
CA19-9: Carbohydrate antigen 19–9
CAF: Cancer-associated fibroblast
CAR: Chimeric antigen receptor
CCSA-2: Colon cancer-specific antigen-2
CD: Cluster of differentiation
CDC25A: Cell division cycle 25A
CEA: Carcinoembryonic antigen
CRC: Colorectal cancer
CSC: Cancer stem cell
DM: Diabetes mellitus
EMT: Epithelial-mesenchymal transition
FBG: Fasting blood glucose
FMN: Flavin mononucleotide
GSEA: Gene set enrichment analysis
HIF-1α: Hypoxia-inducible factor 1α
HK2: Hexokinase type 2
ICOS-L: Inducible costimulatory ligand
IFN: Interferon
IL: Interleukin
IL20RA: Interleukin-20 receptor subunit α
JAK: Janus kinase
KIF15: Kinesin family member 15
LDH: Lactate dehydrogenase
MHC: Major histocompatibility complex
MMP: Matrix metalloproteinase
MSI-L: Microsatellite instability
MSS: Microsatellite stable
MVD: Microvascular density
NF-kB: Nuclear factor kappa light chain enhancer of activated B-cells
NFAT: Nuclear factor of activated T-cells
OS: Overall survival
OXPHOS: Oxidative phosphorylation
PBMC: Peripheral blood mononuclear cell
PBT: Peripheral blood transcripts
PD-L1: Programmed death-1 ligand
PD-L2: Programmed death-2 ligand
PFS: Progression-free survival
PI3K: Phosphoinositide 3-kinase
PLA2R1: Phospholipase A2 receptor 1
STAT: Signal transducers and activators of transcription
TAM: Tumor-associated macrophage
TGF: Tumor growth factor
TIL: Tumor-infiltrating lymphocyte
TLR: Toll-like receptor
TLT-2: TREM like transcript 2
TMA: Tissue microarray
TME: Tumor microenvironment
TNM: Tumor node metastasis
Treg: Regulatory T-cell
TriKETM: Tri-specific killer engager
VEGF: Vascular endothelial growth factor
WTS: Whole tissue section
XRCC1: X-ray repair cross-complementing group
